# Can Immunization of Hens Provide Oral-Based Therapeutics against COVID-19?

**DOI:** 10.3390/vaccines8030486

**Published:** 2020-08-28

**Authors:** José M. Pérez de la Lastra, Victoria Baca-González, Patricia Asensio-Calavia, Sergio González-Acosta, Antonio Morales-delaNuez

**Affiliations:** 1 Biotechnology of Macromolecules Research Group, Instituto de Productos Naturales y Agrobiología, (IPNA-CSIC), 38206 San Cristóbal de la Laguna, Spain; victoria@ipna.csic.es (V.B.-G.); sergi_glez@hotmail.com (S.G.-A.); morales.delanuez@ipna.csic.es (A.M.-d.); 2 Biological Activity Service, Instituto de Productos Naturales y Agrobiología (IPNA-CSIC), 38206 San Cristóbal de la Laguna, Spain; patriciaac@ipna.csic.es

**Keywords:** anti-infective agents, immune therapy, vaccine, antibodies, epitope, peptide

## Abstract

In the current worldwide pandemic situation caused by the Severe Acute Respiratory Syndrome Coronavirus 2 (SARS-CoV-2) and the newest coronavirus disease (COVID-19), therapeutics and prophylactics are urgently needed for a large population. Some of the prophylaxis strategies are based on the development of antibodies targeting viral proteins. IgY antibodies are a type of immunoglobulin present in birds, amphibians, and reptiles. They are usually obtained from egg yolk of hyper-immunized hens and represent a relatively inexpensive source of antibodies. Specific IgY can be produced by immunizing chickens with the target antigen and then purifying from the egg yolk. Chicken IgY has been widely explored as a clinical anti-infective material for prophylaxis, preventive medicine, and therapy of infectious diseases. Administered non-systemically, IgY antibodies are safe and effective drugs. Moreover, passive immunization with avian antibodies could become an effective alternative therapy, as these can be obtained relatively simply, cost-efficiently, and produced on a large scale. Here, we highlight the potential use of polyclonal avian IgY antibodies as an oral prophylactic treatment for respiratory viral diseases, such as COVID-19, for which no vaccine is yet available.

## 1. Introduction

In December 2019, a cluster of patients with pneumonia of unknown cause appeared in Wuhan, Hubei. This new agent was quickly identified as a novel coronavirus, named as SARS-CoV-2 and the disease as COVID-19. COVID-19 is a highly transmissible and pathogenic viral infection. According to national and international agencies responsible for epidemiological surveillance, we face one of the greatest public health problems. Coronavirus is continuing its spread across the world, with more than 23 million confirmed cases of COVID-19, including more than 800 thousand deaths, reported to the WHO. This disease has caused a dramatic healthcare challenge as there is no clinically approved drug or antiviral vaccine against COVID-19. Modelers predict that this coronavirus is here to stay, and we do not know yet whether people develop lasting immunity to the virus [1,2].

The human-to-human spreading of the virus occurs due to close contact with an infected person, exposed to coughing, sneezing, respiratory droplets, or aerosols. These aerosols can penetrate the human body (lungs) via inhalation through the nose or mouth. The virus replicates intensively in the throat and nostrils and later in the lung, causing severe pulmonary lesions. Nasal and sometimes oropharyngeal swabs are used in the polymerase chain reaction test (PCR) to determine the presence of the coronavirus. The protection of the throat and nostrils is therefore critical if we want to stop both the virus dissemination and disease prevention [3].

Vaccines are the gold standard for the control and prevention of infectious diseases, but, in the case of SARS-CoV-2, due to the current absence of specific antiviral drugs, antibodies are an attractive alternative for the development of immuno-therapeutics. Immunity can thus be acquired artificially by active vaccination and passive immunotherapy [4], which will help to recognize and fight a repeat attack by the coronavirus. In addition, antibodies targeting viral proteins can be applied locally in the throat and nostrils to inhibit virus replication. For example, humanized murine monoclonal antibodies against SARS-CoV-2 might be used in a therapeutic antibody cocktail [5]. In another approach, antibodies from camelids, particularly the smaller nanobodies, are being developed for the development of inhalable prophylactic formulations to protect us against this novel coronavirus [6].

Avian antibodies could be a good alternative as they offer several advantages over mammalian antibodies and are cost-effective. In this case, birds, such as hens, are not vaccinated for disease prevention purposes. Instead, they can be hyperimmunized with antigens for the massive production of antibodies. Given that the avian antibodies can be easily collected from the eggs, its production can be scaled up to the industrial fabrication of antibodies for different purposes and biotechnological applications. We here review the use of avian antibodies for the treatment and prevention of viral diseases and propose its potential use for an oral prophylactic treatment for COVID-19.

## 2. Antibody Therapy

The observation that individuals suffering from certain diseases are exempt from re-infection has been well known for a long time. This fact was attributed to the presence of antibodies and is the basis of modern vaccination. When antibodies are transferred from one individual to another, it is referred to as passive immunity [7]. In antibody therapy, the antibodies produce immunity quickly (a few hours), although their effect is not long-lasting (only a few months) because the immune memory is not activated [8].

Artificially induced passive immunity has been used for more than a century to treat infectious diseases, and before the advent of antibiotics. In this type of therapy, we can use antibodies from human serum (homologous) or animals (heterologous). Heterologous antibodies are usually obtained from domestic animals such as horses, rabbits, cows, and goats [9]. Passive transfer is used prophylactically for the neutralization of viruses and toxins and immunodeficiency diseases such as hypogammaglobulinemia. It is also used in the treatment of various types of acute infections and to treat poisoning [10].

Compared to vaccination, passive immunotherapy can be applied at different ages of patients, from infants to adults, including low birth weight or immunodeficient patients or women during pregnancy. However, serotherapy has several drawbacks: its effect is usually short-lived, fading due to the gradual catabolism of the antibodies [11,12]. Therefore, serotherapy is reserved for cases where there is no curative treatment or active induced immunization. The main risk that occurs when the heterologous serum is used is the hypersensitivity reactions, whereas in the homologous serum the danger is the transmission of human diseases, especially hepatitis and AIDS [13,14]. For this reason, the use of polyclonal antibodies as therapeutics has been relegated to other non-systemic applications.

Serotherapy still has valuable applications when there is no effective vaccine, in states of immunodeficiency and cases of multiple infections. The most common is perhaps its use in passive immunization for the treatment of several infections, including tetanus and rabies [15]. We propose that, while waiting for a vaccine to prevent COVID-19 infection, avian heterologous antibodies obtained through so-called IgY technology could be helpful to prevent or treat SARS-CoV-2 infections.

## 3. IgY Technology

In 1983, Klemperer observed that, by immunizing chicks, they produced antibodies, both in their yolks and in blood plasma [16]. Later, it became evident that this was a new type of immunoglobulin present in amphibian, reptile, and birds, named IgY, with the immunoglobulin in birds being the most studied because of its practical nature [17,18]. Birds, unlike mammals, have no colostrum and instead uses the egg yolk as a very effective method of passive transfer of antibodies to their offspring. IgY antibodies can be easily collected and purified by immunizing hens with an antigen of interest. IgY antibodies are thus obtained without the need to bleed the animal [18,19], minimizing suffering and meeting the animal welfare requirements stipulated in the 3Rs principle concerning the refinement. The production of IgY antibodies from poultry eggs is now called IgY technology and is a mature technique that offers numerous applications [18,19,20].

IgY antibodies, extracted from chicken eggs, can be considered a natural product found in a food that is usually part of our diet worldwide. Applied locally and topically, the possible toxicity of prophylactic IgY antibodies should be negligible. In Asian countries, such as Korea and Japan, several products are currently marketed as dietary supplements for the treatment of gastric ulcer due to *helicobacter pylori*. Topical applications of IgY have also been described in the literature to prevent gingivitis or dental caries. IgY antibodies have been administered orally in piglets and calves to prevent diarrhea caused by enterobacteria, in a form of conferring passive immunity. As antimicrobials, IgY antibodies have been orally administered to fishes for the prevention and treatment of several infectious diseases with very promising results. Some systemic uses of IgY have also been proposed in the literature mainly as fast-acting antivenoms [18,19,20].

Other uses of purified IgY antibodies include immobilization on solid supports. For example, air conditioners can incorporate biofilters with specific IgY antibodies to the influenza virus. These biofilters would retain the viral particles in solid support and remove them from the air, thus achieving an air clean of influenza viruses [18,19,20].

## 4. Molecular Properties of IgY

**Figure 1:**
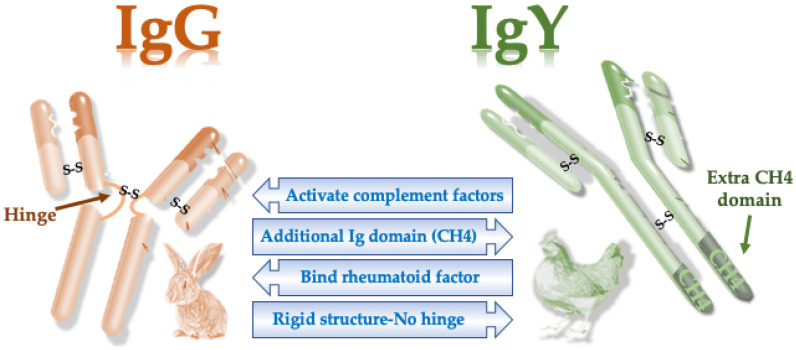
Main structural and functional differences between mammals and avian antibodies. S-S, disulfide bonds.

Similar to mammalian antibodies, IgY is structurally composed of two heavy and light chains. However, there are some molecular differences between mammalian IgG and bird IgY (Table 1). The evolutionary distance between birds and mammals has made these two molecules have different properties [21,22]. For example, unlike IgG, IgY lacks a hinge region between the two “arms” of the antibody (Figure 1). Due to the flexibility provided by the hinge regions, IgY antibodies have a more rigid structure and are somewhat stronger than mammalian antibodies [22,23].

The molecular weight of the light chain is similar in both IgG and IgY, with a value of 25 kDa. However, the heavy chain of IgY has an additional constant domain. Consequently, the size of the avian IgY is larger than mammalian IgG, with a higher molecular weight of its heavy chain (Table 1). The IgY molecule is more hydrophobic than the IgG molecule. Concerning the isoelectric point, IgY is slightly acidic due to the unique side chains of oligosaccharides (Table 1). Antibodies from mammals and birds do not cross-react immunologically [22]. The most important consequence of these molecular differences is that we cannot use protein A or G resins in IgY purification [24] (Table 1).

## 5. Methods for Purification of IgY Antibodies

In general, egg yolk consists of 50% water. The dry matter consists of 2/3 lipids and 1/3 proteins. In particular, egg yolk proteins are distributed between plasma and granular proteins. Granular proteins are composed of α and β lipovitellins (70%), phosvitine (16%), and low-density lipoproteins (12%). On the other hand, plasma proteins consist of α-livetin (serum albumin), β-livetin (2-glycoprotein), γ-livetin (IgY), and low-density proteins [25]. In total, IgY comprises about 3–5% of egg yolk proteins dispersed in a yolk lipid emulsion, together with lipoproteins and glycoproteins. All livetin are water-soluble. That is why one of the problems of large-scale separation of IgY antibodies consists of separating water-soluble proteins from lipids and other hydrophobic substances [25].

Some of the most common methods published in the scientific literature consist of the removal of the lipid portion, and subsequent fractionation of the protein portion, using several chemical substances [26]. For example, the aqueous dilution method is based on adding water to the egg yolk to separate the lipid portion of the aqueous fraction, leaving the IgY antibody in the water-soluble fraction (WSF) [27]. This method has undergone slight modifications, such as the use of acidic water, adjusted to pH 5, and cold-treatment, which may consist of refrigeration or freezing before fractionation [28].

Another of the most commonly used IgY extraction methods is the differential precipitation of egg yolk proteins with PEG [29,30,31,32] or natural gums, such as xanthan or carrageenan [33].

## 6. Advantages and Disadvantages of Avian IgY over Mammalian IgG

There are several advantages of using chicken antibodies instead of mammalian antibodies (Table 2). For example, it may be advantageous for certain applications that IgY antibodies are not able to activate the complement system [34]. In addition, the large phylogenetic distance between mammals and birds would allow IgY antibodies to recognize certain mammalian epitopes, which might not be recognized efficiently if mammalian antibodies were used [35,36,37]. One of the major limitations of IgY most likely lies in their purification from egg yolks. Unfortunately, a procedure as simple as the preparation of antisera from mammalian blood is not available for chicken antibodies [38].

When applied as antimicrobial, microorganism-specific polyclonal IgY antibodies offer the advantage that they are not able to induce resistance, because they usually recognize multiple epitopes on the exposed surface, which is the consequence of the expression of many genes. Moreover, these antibodies, being highly specific, are directed only towards the target microorganism, without affecting the rest of the bacteria present in the normal bacterial flora. In this sense, antimicrobial antibodies are considered a good alternative to the use of antibiotics due to their higher selectivity and their origin, as a natural product that respects the environment and the principle of the 3Rs [39].

The advantages of using passive immunotherapy based on IgY antibodies instead of serum or monoclonal antibodies (or fragments of these) are that IgY is cheap to produce and it is easy to obtain desired samples. For example, 30 days of IgY production can easily reach values greater than 2 g.

Furthermore, these antibodies can be administered orally with total safety and are well tolerated in humans. IgY antibodies can be purified, stored for long periods even at room temperature, and formulated to provide rapid passive protection [40,41]. Therefore, IgY treatments are ideal for combating a pandemic since they can be manufactured and stored worldwide using commercially available laying hens that are available in every country that can produce them [42,43,44,45].

## 7. The Production Capacity of IgY Antibodies

One of the most important benefits of IgY technology is the low cost of the antibodies and the high yield obtained when the antibodies are purified from the egg yolk. If we compare the equivalent amount of antibody taken from the blood of a small mammal, such as the rabbit, IgY antibodies are superior. For example, taking into account that an average egg contains about 60 mg of IgY, a single hen laying about 320 eggs per year could produce about 20 g of antibody [32]. With 50 hens, 1 kg of IgY could be produced in one year. The maintenance cost of the hens is also much lower than the cost of maintaining the farm animals if milk or cow’s blood is used as the source of IgG [46]. In addition, hens are better antibody producers than rabbits, because they produce higher levels of antibodies with fewer immunizations [47,48]. In competition assays, it has been shown that IgY recognizes their antigens with an affinity five times higher than the corresponding mammalian IgG [49].

Concerning the collection of antibodies, IgY immunoglobulins are easily collected from the egg-laying, without the need to bleed the animals [50]. This is not the case for mammalian IgG antibodies, which are collected by blood extraction causing stress and pain to the animals. Besides, IgY antibodies can be kept for a certain time without the need for refrigeration, e.g., in the form of freeze-dried powder they can be kept at room temperature with a minimum of humidity. These advantages make IgY antibodies ideal for production in various countries of the world, regardless of their level of development. In this respect, IgY antibodies for therapeutic or prophylactic purposes offer a good alternative to the control of pandemics and bioterrorist attacks. For example, IgY antibodies to influenza viruses, obtained from ostrich eggs, have been proposed for the control of the pandemic influenza A H1N1 virus. It has been suggested that these antibodies could be applied to solid supports such as masks and filters to avoid inhalation of the virus [51]. The same scenario could be applied for the control of SARS-CoV-2 with the attachment of specific IgY to such solid supports.

## 8. Stability of IgY

IgY has demonstrated good stability to pH and thermal treatments, which greatly facilitates its application for both human and veterinary purposes. The stability of these antibodies against changes in pH, temperature, salt concentration, and protease degradation is a very important aspect when considering the possible biotechnological applications offered by IgY technology [40,41,52,53].

Due to their proteinic nature, IgY antibody preparations were considered unstable and easily digestible. However, numerous studies showed the efficiency of orally administered IgY against infectious agents in the food tract from the oral cavity to the intestines [54,55,56]. Several authors have reported that IgY antibodies are stable at acid pH, above pH 3 values. At alkaline pH values, up to 11, no changes in the activity of these IgY antibodies have been reported, although a loss of efficacy at pH values above 12 has been demonstrated [39,57,58].

The binding capacity of IgY to its antigens appears to stably withstand temperatures up to 60–70 °C, but it is lost at temperatures above 75 °C. On the other hand, IgY remains stable at 4 °C for more than five years and one month at 37 °C, with hardly any loss of activity [58,59].

Regarding the stability of IgY antibodies against proteases, it appears that they are sensitive to pepsin digestion, particularly at pH values below 5, but moderately resistant to trypsin chymotrypsin digestion [60]. For example, it has been shown that after 8 h of protease digestion about 40% of the IgY molecule remained intact and functional. IgY is therefore thought to resemble secretory IgA, a protease-resistant immunoglobulin that is naturally secreted in the intestinal lining of mammals for mucosal protection [17].

Although IgY is susceptible to degradation by pepsin under acidic conditions, it is now possible to overcome this difficulty by suitable enteric coating formulations or delayed-release capsules and to protect the immunoglobulin from attack by hydrochloric acid in the stomach. IgY antibodies can be wrapped in liposomes, gelatin or alginate microcapsules or attached to nanotubes to increase its stability and assimilation in the right place. It is also possible to achieve an acid-buffering effect by co-administering IgY with certain foods, such as milk and egg protein [61,62].

Therefore, due to their pH and proteolysis stability, IgY antibodies are very useful for passive immunization therapy through oral ingestion. Freeze-dried immunoglobulin preparation may be administered orally as a nutraceutical or food additive [39].

## 9. Virus Protection Induced by IgY Antibodies

**Figure 2:**
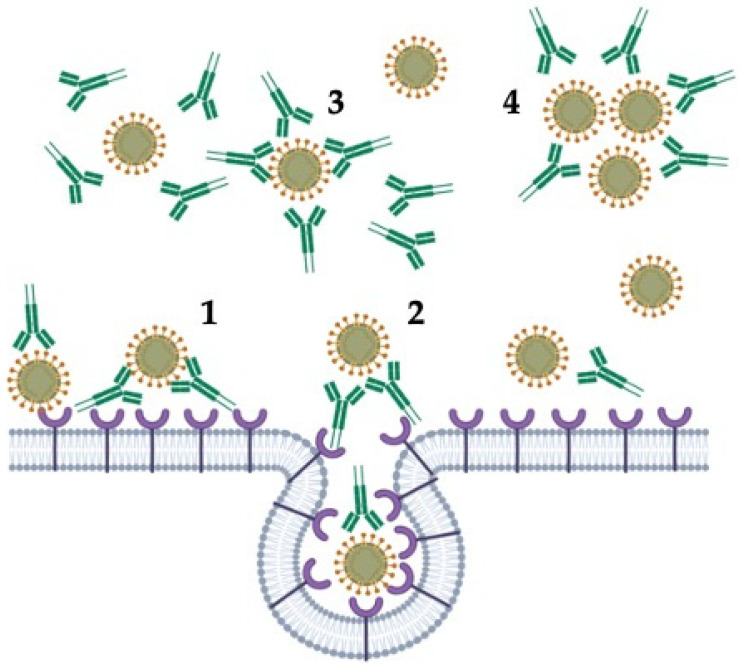
Mechanisms of inhibition of viral infectivity by antibodies. (**1**) blocking the attachment of the virus to host tissue; (**2**) preventing the membrane fusion or promoting the detachment of bound virus; (**3**) interfering with free virions; and (**4**) causing aggregation of virus particles with resulting virus immobilization (Created with BioRender.com).

Upon viral infection of vertebrates, antibodies are produced against many epitopes on multiple virus proteins. Antibodies can neutralize viral infectivity by several mechanisms [63]: (1) blocking the attachment of the virus to host tissue; (2) preventing the membrane fusion or promoting the detachment of bound virus; (3) interfering with free virions; and (4) causing aggregation of virus particles, acting as a “biological glue” with resulting virus immobilization (Figure 2).

Homolog antibodies, together with serum complement, may disrupt viral membranes, whereas non-neutralizing antibodies, which bind specifically to virus particles but do not neutralize infectivity, could inhibit the activity of key enzymes [63,64,65].

Other non-neutralizing antibodies can also be produced after viral infection, but, instead of preventing intercellular spread, such antibodies may enhance infectivity by interacting with Fc receptors on macrophages. This antibody-dependent enhancement (ADE) mechanism may allow the entry of the virus into cells that normally do not bear specific virus receptors [65]. Given that a key feature of IgY is the lack of interaction with mammalian or known bacterial FcγR or Fc binding receptors, IgY antibodies will not induce ADE when encountering subsequent serotypes of viral antigens.

Several studies have demonstrated the efficacy of the use of IgY in viral infections (Table 3). Passive immunization experiments in mice have shown that intranasal administration of IgY antibodies against the H5N1 influenza virus conferred protection against a lethal infection of a highly pathogenic H5N1. Besides, these anti-H5N1 antibodies also conferred protection against the H1N1 virus strain, demonstrating the protective capacity of polyclonal antibodies, despite the differences between different strains of the influenza virus. In addition, these mice developed a protective immune memory which makes the use of IgY antibodies used in passive immunization even more practical [44,64,66].

Rotaviruses cause severe diarrhea disease in young children around the world. According to the WHO’s 2008 estimates, about 450,000 children under the age of five die each year from this disease. In the case of rotavirus that causes diarrhea in infants and young children, several clinical trials have demonstrated the utility of an oral preparation based on IgY anti-rotavirus antibodies (Rotamix) in the control of this infectious disease. Despite the availability of vaccines, the IgY preparation proved to be a promising, safe, and effective supplement for the treatment of acute diarrhea in pediatric patients [67].

The antiviral effect of IgY antibodies was also studied with flaviviruses, such as dengue virus (DV), West Nile (WNV), and Zika virus (ZV) [68,69,70,71]. Dengue fever is a viral infection transmitted by mosquitoes of the species *Aedes aegypti* and, to a lesser extent, *A. albopictus*. These mosquitoes also transmit chikungunya, yellow fever, and Zika virus infection. According to a recent estimate, there are 390 million dengue infections each year, resulting in 500,000 cases of dengue hemorrhagic fever and 22,000 deaths. The severity of flavivirus infection is greatly enhanced by antibody-dependent enhancement (ADE). Administered in mice, goose IgY antibodies against DV, WNV, and ZV were effective in preventing the corresponding infection without inducing in vitro ADE [68]. These antibodies were also found to recognize epitopes on the envelope, membrane, and nonstructural proteins of the virus, confirming the potency of the polyclonal response that can be obtained with this IgY technology [68,69,70,71].

Hantaviruses are emerging zoonoses hosted by small mammals. In humans, they cause two diseases: hemorrhagic fever with renal syndrome and cardiopulmonary syndrome. Goose derived IgY generated against a DNA vaccine of hantavirus produced a robust humoral response. In vivo experiments showed that Andesvirus-specific IgY protected from Hantavirus pulmonary syndrome in a hamster model [72,73].

IgY antibodies have been tested against rabies. Chickens immunized with a recombinant part of the G protein of rabies virus neutralized virus infectivity in vitro. Moreover, the inoculation of the antibody into mice infected with rabies virus reduced the mortality caused by the virus [74,75,76,77,78].

For Severe Acute Respiratory Syndrome (SARS), high-titer anti-SARS IgY was obtained by immunization of pathogen-free chickens with inactivated SARS coronavirus. In vitro, the IgY antibody effectively neutralized the SARS coronavirus until 1:640 dilution, indicating its potential use for passive immunization [42].

The infection with Norovirus (NV) causes gastroenteritis, an inflammation of the stomach and intestines. A norovirus is a group of viruses that belong to the family *Caliciviridae*. Their genome comprises a single positive RNA chain and they are transmitted by the fecal–oral route. Each year, NV causes 685 million cases, no specific medication is available for its treatment, and no specific vaccine is available to prevent NV infection. In in vitro infection experiments, polyclonal IgY antibodies against NV P-particles showed infection-blocking activity. This opens up the possibility of obtaining a treatment based on IgY antibodies [79,80].

The Ebola virus (EBV) is a genus of the virus of the family Filoviridae that was first detected in some regions of Africa. This virus is a very virulent pathogen that causes hemorrhagic fever with high mortality rates in humans. The infection is transmitted by direct contact with the blood, body fluids, and tissues of infected animals or people. Experimental assays with IgY antibodies showed blocking activity in vitro. Besides, newborn mice that received a passive transfer of IgY antibodies were shown to achieve complete protection against a lethal dose of viral challenge [59].

In summary, these studies demonstrate the utility of IgY in the control of viral infections. Most of these studies showed that the use of IgY conferred in vitro and/or in vivo protection without producing ADE. Moreover, the utility of IgY antibodies has been validated even when vaccines are available for a particular viral disease. In some instances, the polyclonal response obtained by IgY antibodies was also effective against multiple epitopes and subunits of the exposed virus.

## 10. Peptide-Based Immunization against SARS-CoV-2

In the current pandemic situation caused by the SARS-CoV-2, therapeutics, and prophylactics are urgently needed for a large population. We here propose that avian IgY antibodies are useful for the development of such therapeutics and prophylactics. To make the immune response to IgY antibodies even more specific, one common strategy consists of peptide-based immunization, rather than using live or attenuated SARS-CoV-2 virus. When peptides are used as antigens, instead of whole proteins, the antibodies that are generated recognize a small portion of the antigen, called an epitope. These epitopes can be linear or conformational, depending on the arrangement of their residues in space and along with the three-dimensional structure of the protein [81]. One of the most commonly used proteins for peptide immunization is the Spike viral protein. This protein interacts with the human receptor of angiotensin-converting enzyme 2 (ACE2) and allows the entry of the virus into host cells. Therefore, one of the strategies in the development of protective antibodies against SARS-CoV-2 is to know the contact regions between the Spike protein and ACE2 and to generate antibodies against these critical epitopes of this protein in the hope that these antibodies will block the entry of the virus and prevent the development of the disease [82]. Peptide-based immunization in birds would guarantee the safety of this particular type of vaccination, not aimed at disease prevention, but at the mass production of polyclonal antibodies [83,84,85].

The bioinformatic approach is an effective initial step to screen potential B-cell linear epitopes of SARS-CoV-2. These predicted epitopes, however, need experimental verification to confirm their immunogenicity and neutralization abilities. Bioinformatic and computational tools are involved in the theoretical prediction of critical epitopes. Once the structure of the antigens is known, it is possible to make calculations and predictions that lead to the analysis of the possible epitopes that trigger an immune response. These powerful bioinformatic tools are commonly used in vaccine design and the development of therapeutic antibodies for numerous clinical and biotechnological applications. It is important to consider that peptides alone are generally too small to elicit a satisfactory immune response. Usually, to produce antibodies against peptides, synthetic peptides are coupled to carrier proteins to facilitate the induction of the B-cell response. Keyhole limpet hemocyanin (KLH) and bovine serum albumin (BSA) are two major kinds of carrier proteins to generate anti-peptide antibodies [86,87].

## 11. IgY as Promising and Safe Oral Treatment

**Figure 3:**
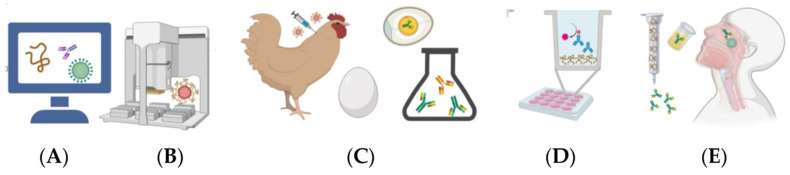
Steps for the development of an oral-based therapeutic: (**A**) epitope analysis; (**B**) peptide synthesis coupled to a carrier protein; (**C**) hens immunization and antibody collection; (**D**) immunoassay for a screening of specificity; and (**E**) enrichment and final development of an oral-based therapeutic gargle. Created with BioRender.com.

Active immunization or vaccination depends on the host’s immune system to generate an immune response that is usually delayed for several days or a few weeks. In contrast, the use of IgY as a prophylactic treatment for a topical and local user has the advantage that its effect is almost instantaneous [88]. The development of an oral-based therapeutic formulation against COVID-19 involves several steps: from the study of the B-cell epitopes found in viral proteins to the development of a gargle containing neutralizing IgY antibodies (Figure 3). When administered locally, the neutralizing or binding effect of IgY may inhibit virus colonization and replication in the mucous membranes by losing their ability to adhere to them, preventing damage to the mucosal lining, while the systemic and local immune systems elaborate their protective antibodies to eliminate the pathogens and prevent the onset of disease [75].

Heat treatments such as pasteurization, sterilization, or spray drying of IgY is critical to ensure the stability of IgY antibodies in an oral formulation or during product storage. Some previous work has studied this aspect and concluded that IgY passed the pasteurization process being suitable for human consumption. For example, IgY antibodies to human noroviruses are stable at temperatures below 70 °C. In other in vitro functional tests, IgY antibodies subjected to 75 °C were found to be incapable of showing blocking activity, suggesting that IgY could be denatured at this temperature [88,89,90,91,92,93,94]. To confer stability to IgY antibodies in oral formulations, it has been shown that the addition of sugars, glycerol, glycine, or gum arabic coatings to antibody solutions was able to protect IgY and reduce the degree of hydrolysis [52,60].

Compared to mammalian or camelid antibodies, IgY antibodies obtained from egg yolk are ideal for use as part of an oral formulation for human consumption. IgY antibodies are derived from a natural product, such as the egg, with good social acceptance, in contrast to the other types of antibodies, which are obtained purified from the blood of animals or as recombinants products from the microbial culture [92,93,94]. Concerning the ability of antibodies to induce cell activation or produce inflammation, bird IgY antibodies are superior, since their Fc portion is not capable of activating the human complement system, nor reacting with rheumatoid factors or human Fc-receptors [95].

The mouth is the portal of entry for many infectious agents, such as the novel coronavirus SARS-CoV-2. Taken orally, uncoated IgY antibodies are completely safe, since they would be destroyed in the stomach, cannot reach the intestine and pass into the blood unless they have been previously vehiculated somehow for this purpose. Since they are specific to a particular virus, pathogen, or epitope, oral treatment with this type of antibody would not generate resistance or alter the normal bacterial flora [92]. With recent consumer trends in favor of natural products to alleviate health problems, oral IgY antibody-based preparations for COVID-19 prophylaxis would be well accepted by society. Since IgY is found in the egg yolk, it would be comparable to eating raw egg yolk as long as the subject is not allergic to eggs [92,96,97].

The efficacy and safety of an oral formulation based on IgY antibodies have been proven in patients with cystic fibrosis (CF) [98]. CF is characterized by excessive and persistent inflammation in the airways in response to bacterial infections that destroy the lungs and decreased lung function. In a study approved by the Swedish Medical Products Agency and the Ethics Committee of Uppsala University Hospital, oral therapy with IgY anti-Pseudomonas was evaluated as prophylaxis for patients with CF [99]. With this treatment, IgY would form an antibody barrier that prevents *P. aeruginosa* from entering the lungs through the nose and oropharynx and binding to the epithelial surface of the mucosa, thus preventing the invasion of bacteria into the lower respiratory tract. The study showed that, when gargling with IgY after brushing teeth at night, active concentrations of Anti-*Pseudomonas* IgY were present in saliva and oropharyngeal mucosa the next morning [100].

With a similar approach, an oral formulation based on neutralizing IgY antibodies may block the entry of SARS-CoV-2 virus into the mouth, where it actively replicates, and prevent the development of disease or injury to the lungs (Figure 3). Generally, the severity or extent of clinical pathologies is directly proportional to the magnitude of the bacterial or viral load. Therefore, such oral formulation could decrease the effective viral load and thus enable susceptible individuals to mount an adequate immune response by themselves. This type of oral treatment would also be very useful in immunocompromised individuals, such as the elderly, children, those infected with HIV, or the sick and debilitated, especially cancer patients [101,102,103].

Apart from the conceptualization of the utility of these antibodies for COVID-19, IgY antibodies have been marketed as oral formulations and foods in the form of yogurt, pills, tablets, capsules, milk formulas, etc. against dental caries, gingivitis, oral thrush, infantile rotavirus, and gastric ulcer [103]. As a drug, an oral formulation of IgY (gargle) against *P. Aeruginosa* has been attempted to be registered for cystic fibrosis [100]. Other formulations, such as tablets or lozenges, have been proposed for a powdered form of IgY since it is highly stable at room temperature for at least two years [103].

## 12. Conclusions

Humanity is facing one of the greatest health challenges accompanied by great economic losses. In a scenario where vaccination against COVID-19 is not yet available to everyone or does not yet offer adequate safety guarantees, the search for other prophylactic treatments becomes necessary. Many prophylactic treatments, including the desirable vaccines, are based on the development of host antibodies since they are proteins with high specificity and capable of neutralizing the virus or helping to eliminate it [104,105]. Currently, more than a hundred projects are being developed to achieve a COVID-19 vaccine [106,107], although the WHO warns that this may never come. One of the main problems is that the antibodies induced by vaccines may not be protective, the humoral response could be deficient, or they would not generate memory, making it possible that the same individual could be re-infected several times throughout his life [107].

There is an inevitable time lag between the start of a pandemic and the ability of the clinical community and pharmaceutical and biotechnology companies to respond. Therefore, until an effective vaccine is widely available, the global effort to prevent SARS-CoV-2 infections will have to continue for some time. IgY antibodies from chicken egg yolk could be a good alternative because of their viability for large-scale commercial production and the relative non-invasive methods used to prepare them [44,108,109]. Clinical and laboratory data demonstrate that IgY is a safe and effective tool for the control of viral diseases and it can be used as a substitute for, or as an essential complement to, antimicrobials or vaccines against certain viruses and respiratory pathogens [96]. While IgY may not eradicate target microbes, it could significantly reduce infectious pathogen load to a point where an antimicrobial agent, or in most cases the host’s own immune system, can more easily subjugate the invading pathogen [110,111]. Likewise, another IgY-based immune interventions, the development of IgY antibodies with peptide-based immunization of hens, may provide oral-based therapeutics and prophylactics against this high priority coronavirus (Figure 3). For example, it could be a remarkable success if an effective reduction of SARS-CoV-2 viral load is achieved in the upper respiratory tract [105,112]. The use of chickens instead of other farm animals in the production of these antibodies allows industrial scale-up and its use in many countries worldwide where chicken farming is available.

## Figures and Tables

**Table 1:** Molecular features of mammalian and avian antibodies.

Molecular Feature	Mammalian IgG	Avian IgY
Molecular weight	150 kDa	180 kDa
No. of constant domains	4	3
Isoelectric point	6.1–8.5	5.7–7.6
Hinge region	Present	Absent
Complement binding	Present	Absent
Rheumatoid factor binding	Present	Absent
Mediates anaphylaxis	No	Yes
Binding to protein A or G	Yes	No

**Table 2:** Advantages and disadvantages of IgY polyclonal antibodies.

Advantages	Disadvantages
Easy to produce and easily sampled	Mixed with other non-specific antibodies
Low cost, less invasive, a higher amount	Less used in the pharmaceutical industry
Can identify various epitopes on a given antigen	Cannot be purified by protein A or G
Detect small changes within antigens	Risk of different efficacy among batches
Remain stable at variable pH and temperatures	-
Can be produced in large batches	-
Can be safely used in the food industry	-
Perform better recognition of conserved mammalian epitopes than IgGs	-
No cross-reactivity with rheumatoid factors	-
No activation of the mammalian complement	-
High resistance to immunization with toxins	-

**Table 3:** Efficacy of the utilization of IgY in viral infections.

VIRUS	Model	Antigen	Effect	Reference
**INFLUENZA A**	Mice	whole inactivated viruses	In vivo protection against lethal challenge with H5N1	[44,64,66]
**ROTAVIRUS**	Young children	Whole virion particle	Reduced the severity of clinical manifestation of diarrhea among IgY-treated subjects	[67]
**DENGUE**	Mice	Dengue Type 2 Antigen	Neutralizing but not enhancing virus infection	[68,69,70]
**ZIKA**	Mice	Inactivated PRVABC59 Zika virus	In vitro inhibition without inducing ADE	[71]
**HANTAVIRUS**	Hamster	M segment-DNA vaccine	In vivo efficacy after intranasal challenge	[72,73]
**RABIES**	Mice	Recombinant rG-F2 protein	Neutralized rabies virus infectivity	[74,75,76,77,78]
**SARS**	In vitro binding assay to Vero E6 cells	Inactivated SARS virus strain BJ01	In vitro neutralization	[42]
**NOROVIRUS**	In vitro binding and blocking assay	Recombinant P particles	In vitro blocking activity	[79,80]
**EBOLA**	Mice	Recombinant antigens	In vivo protection	[59]
